# Phrenic nerve stimulation mitigates hippocampal and brainstem inflammation in an ARDS model

**DOI:** 10.3389/fphys.2023.1182505

**Published:** 2023-05-05

**Authors:** Thiago G. Bassi, Elizabeth C. Rohrs, Karl C. Fernandez, Marlena Ornowska, Michelle Nicholas, Jessica Wittmann, Matt Gani, Doug Evans, Steven C. Reynolds

**Affiliations:** ^1^ Lungpacer Medical Inc., Vancouver, BC, Canada; ^2^ Advancing Innovation in Medicine Institute, New Westminster, BC, Canada; ^3^ Fraser Health Authority, Royal Columbian Hospital, New Westminster, BC, Canada; ^4^ Biomedical, Physiology, and Kinesiology Department, Simon Fraser University, Burnaby, BC, Canada

**Keywords:** mechanical ventilalion, brainstem inflammation, ARDS, neuroinflammation, phrenic nerve stimulation

## Abstract

**Rationale:** In porcine healthy-lung and moderate acute respiratory distress syndrome (ARDS) models, groups that received phrenic nerve stimulation (PNS) with mechanical ventilation (MV) showed lower hippocampal apoptosis, and microglia and astrocyte percentages than MV alone.

**Objectives:** Explore whether PNS in combination with MV for 12 h leads to differences in hippocampal and brainstem tissue concentrations of inflammatory and synaptic markers compared to MV-only animals.

**Methods:** Compare tissue concentrations of inflammatory markers (IL-1α, IL-1β, IL-6, IL-8, IL-10, IFN-γ, TNFα and GM-CSF), pre-synaptic markers (synapsin and synaptophysin) and post-synaptic markers (disc-large-homolog 4, N-methyl-D-aspartate receptors 2A and 2B) in the hippocampus and brainstem in three groups of mechanically ventilated pigs with injured lungs: MV only (MV), MV plus PNS every other breath (MV + PNS50%), and MV plus PNS every breath (MV + PNS100%). MV settings in volume control were tidal volume 8 ml/kg, and positive end-expiratory pressure 5 cmH_2_O. Moderate ARDS was achieved by infusing oleic acid into the pulmonary artery.

**Measurements and Main Results:** Hippocampal concentrations of GM-CSF, N-methyl-D-aspartate receptor 2B, and synaptophysin were greater in the MV + PNS100% group compared to the MV group, *p* = 0.0199, *p* = 0.0175, and *p* = 0.0479, respectively. The MV + PNS100% group had lower brainstem concentrations of IL-1β, and IL-8 than the MV group, *p* = 0.0194, and *p* = 0.0319, respectively; and greater brainstem concentrations of IFN-γ and N-methyl-D-aspartate receptor 2A than the MV group, *p* = 0.0329, and *p* = 0.0125, respectively.

**Conclusion:** In a moderate-ARDS porcine model, MV is associated with hippocampal and brainstem inflammation, and phrenic nerve stimulation on every breath mitigates that inflammation.

## Introduction

Acute respiratory distress syndrome (ARDS) is, unfortunately, common in critically ill patients (Fan et al., 2017; Rezoagli et al., 2017). Preclinical studies have shown an association between ARDS and hippocampal apoptosis and inflammation (van Munster et al., 2011; Chen et al., 2015; Chen et al., 2016; Kamuf et al., 2018). Mechanical ventilation (MV) has recently been associated with hippocampal apoptosis and inflammation, also called ventilation-associated brain injury (VABI) (Bassi et al., 2020; Bassi et al., 2021a; Bassi et al., 2021b; Bassi et al., 2021c; Bassi et al., 2022a; Rohrs et al., 2022).

Our group recently reported that in a porcine model of moderate ARDS, MV for 12 h resulted in hippocampal apoptosis and neuroinflammation (Bassi et al., 2022a; Bassi et al., 2022b). The magnitude of the hippocampal apoptosis and inflammation was comparable to the magnitude of these same phenomena observed in pigs without ARDS, mechanically ventilated for 50 h (Bassi et al., 2022a; Bassi et al., 2022b). Although cognition was not measured in these preclinical studies, there is clinical relevance to these findings because a post-mortem clinical study showed an association between hippocampal inflammation and delirium during hospitalization (van Munster et al., 2011).

Previously our group has shown that phrenic nerve stimulation (PNS) as an adjunct to MV resulted in neuroprotection after 50 h of MV alone, and after 12 h of MV after moderate ARDS (Bassi et al., 2021b; Bassi et al., 2021c; Bassi et al., 2022a; Rohrs et al., 2022). These preclinical studies only studied and reported neuroprotection in the hippocampus (Bassi et al., 2021b; Bassi et al., 2021c; Bassi et al., 2022a). It can be argued that other brain areas may also have been affected by the inflammation consequent to ARDS and MV (Quilez et al., 2011). The brainstem is a crucial area that sets the pace for respiration (Nair et al., 2017). The pre-Bötzinger complex in the brainstem sends neural signals to the diaphragm via phrenic efferent neurons, causing diaphragmatic contractions, and receives a sensory signal from the diaphragm via phrenic afferent neurons (Nair et al., 2017). So, during the respiratory cycle the brainstem is in constant communication with the diaphragm via the phrenic nerves (Nair et al., 2017). Preclinical studies have reported that the presence of inflammation in the brainstem is linked to reduced breathing variability and increased work of breathing (Jacono et al., 2011; Sheikhbahaei et al., 2018). Although our group demonstrated that PNS mitigated hippocampal inflammation and cellular apoptosis associated with MV and moderate ARDS, the neuroprotection was studied and measured only in the hippocampus, thereby limiting our findings to one brain area (Bassi et al., 2022a). To extend our previous findings of VABI mitigation, we conducted a *post hoc* analysis to investigate the effects of MV with moderate ARDS on the brainstem and the potential neuroprotection provided by PNS. Our group also expanded the previous analysis in the hippocampus by measuring, *post hoc*, inflammatory proteins in the hippocampal tissue. There is a vast amount of published literature showing a link between low concentrations of synaptic markers and reduced cognitive function (Sze Chun et al., 1997; Meng et al., 2022). Our group therefore also analyzed, *post hoc*, the tissue concentrations of some synaptic markers in the hippocampus and the brainstem in this study.

## Methodology

### Animals

This study received approvals UBC Ethics Committee and Animal Care Committee approvals (ethics certificate #A20-0245 & Approval date 05-Jan-2022) and it followed local Animal Care Committee guidelines. (Bassi et al., 2021b; Rohrs et al., 2021; Bassi et al., 2022a). A porcine model (Yorkshire pigs ranging from 4 to 5 months old) was chosen based on anatomical similarities with humans. Our experiment studied only female pigs due to their availability in the animal facility used for the experiments.

## Experimental protocol

Three groups were studied: mechanically ventilated with injured lungs (MV); mechanically ventilated with injured lungs plus PNS every other breath (MV + PNS50%), and mechanically ventilated with injured lungs plus PNS every breath (MV + PNS100%). All pigs were ventilated for 12 h post-injury. A central line catheter was inserted in the left subclavian vein of all animals included in this study.

During the experiments, our group closely monitored the mean arterial pressure, arterial blood gases, glucose levels, temperature, and heart rate, ensuring that the values were within normal ranges.

### Mechanical ventilation

An Evita XL, Dräger ventilator was used to ventilate the animals. All pigs were ventilated in volume control mode to ensure that the tidal volume delivered was the same in all pigs from all groups. The ventilatory settings followed the lung protective guidelines; 5 cmH_2_O positive end-expiratory pressure, 8 ml/kg tidal volume, respiratory rate ranging from 18 to 22 breaths per minute, trigger threshold of 3l/min, and FiO_2_ of 0.30 to maintain SpO_2_ > 92%. Eight millilitres per kilogram was used because of the greater dead space observed in pigs (the distance from the oral cavity to the lungs). (Grasso et al., 2007). A normal arterial blood gases were achieved by changing the respiratory rate and the fraction of inspired oxygen when necessary.

An esophageal catheter was inserted to measure the esophageal and gastric pressure in order to collect the ventilatory physiological data from the first and last hour of the experiments (FluxMed GrT, MBMed). Transpulmonary plateau pressure, plateau pressure, driving pressure, and esophageal pressure were collected. Esophageal pressure was measured throughout the breath. Plateau, driving and transpulmonary plateau pressures were all measured during an end-inspiratory pause.

The formula, end-inspiratory transpulmonary plateau pressure minus end-expiratory plateau pressure, was used to calculate transpulmonary driving pressure. Real-time ventilator pressure-time product was obtained by respiratory monitoring using a respiratory monitor (FluxMed GrT, MBMed).

### PaO_2_/FiO_2_ ratio

Arterial blood gas samples were used to calculate PaO_2_/FiO_2_ ratio. Samples were taken at baseline, every 4 h, and as clinically indicated.

### Lung injury

Moderate ARDS was induced by injecting cis-9-Octadecenoic acid into the pulmonary artery via Swan-Ganz catheter (via distal port). Between 0.1 and 0.4 ml of cis-9-Octadecenoic acid mixed with the animal’s fresh blood was injected into the pulmonary artery. Moderate ARDS was achieved when the PaO_2_/FiO_2_ ratio was greater than 100 mmHg and lower than 200 mmHg (Grasso et al., 2007).

## Diaphragm contractions and diaphragm contribution with PNS

A central venous catheter embedded with electrodes (LIVE^®^ Catheter, Lungpacer Medical Inc.) was utilized to stimulate bilaterally the phrenic nerves, resulting in diaphragm contraction in synchrony with the inspiratory phase of MV. Diaphragm contractions were synchronized with the ventilator’s inspiratory phase, targeting a ventilator pressure-time-product reduction of 15%–20%. Ventilator pressure-time product was monitored in real-time via FluxMed GrT MB Med, keeping the ventilator pressure-time-product within the targeted range (Bassi et al., 2021b; Rohrs et al., 2021). Phrenic nerve stimulation was achieved by a series of electrical pulses in a stimulation train, at the onset of inspiration, for the duration approximately equal to or slightly less than the inspiratory time set on the ventilator. For example, if the inspiratory time set was 1 s, the duration of the pulses was 900 ms. The stimulus pulses were delivered at a frequency of 40 Hz. The pulse width of the pulses was modulated within the stimulation train ranging between 200 and 300 microseconds.

### Hippocampal and brainstem sampling and preparation

At the end of the study, the hippocampus and the brainstem were harvested for analysis. As previously reported, hippocampal slides were also stained for terminal deoxynucleotidyl transferase-mediated dUTP nick-end labeling (TUNEL), glial fibrillary acid protein (GFAP), and ionizing calcium-binding adaptor molecule-1 (IBA-1) markers to quantify the percentages of positively-stained cells as a proportion of the total cell population for each marker. These data (positively-stained cell percentages) have been published previously, and were used here only to conduct a correlation analysis (Bassi et al., 2021b; Rohrs et al., 2021).

As part of the *post hoc* analysis, an independent laboratory (Wax-it Histology Services Inc.), blinded to sample groups, performed hippocampal and brainstem tissue homogenization. A second independent laboratory (Eve Technologies), also blinded to group allocation, performed and processed enzyme-linked immunoassays for inflammatory and synaptic markers in the homogenized tissue. In the *post hoc* analyses, the inflammatory markers analyzed were: interleukin-1α (IL-1α), interleukin-1β (IL-1β), interleukin-6 (IL-6), interleukin-8 (IL-8), interleukin-10 (IL-10), tumor necrosis factor-α (TNFα), granulocyte macrophage-colony stimulating factor (GM-CSF), and interferon-γ (IFN-γ).

Synaptic markers (presynaptic and post-synaptic) were also analyzed in both the hippocampus and brainstem. Presynaptic markers analyzed were synaptophysin, and synapsin (Mirza and Zahid, 2018; Meng et al., 2022). In clinical studies, lower concentrations of synaptophysin and synapsin have been associated with cognitive impairment (Mirza and Zahid, 2018; Meng et al., 2022). Post-synaptic markers analyzed were disc-large-homolog 4 (DLG4), N-methyl-D-aspartate receptor 2A (NR2A) and N-methyl-D-aspartate receptor 2B (NR2B). In preclinical studies, these three post-synaptic markers have been associated with cell survival and apoptosis (Martel et al., 2009).

### Statistical analysis

Non-parametric tests were utilized, and included either the Kruskal-Wallis test and Dunn’s multiple comparison test, or the Spearman correlation test, as appropriate. Data are expressed as the median and interquartile range (IQR) unless otherwise stated. *p*-values ≤0.05 are considered statistically significant. Statistical analyses used Prism 8.4.2 software (GraphPad).

The ratios of cleaved to complete poly (ADP-ribose) polymerase 1, which is a marker for apoptosis, was used to perform the power calculation (data from González-López, et al., 2013): where the control group had a mean of 1.0, with a standard deviation ± 0.2 and the mechanically ventilated group had a mean of 6.0, with a standard deviation ± 0.8. The power calculation used an α = 0.05 and β = 0.90. (González-López et al., 2013). The calculation indicated that one subject per arm would be sufficient to achieve statistical power. As this would pose a risk of bias due to the small sample size required and one subject per arm would not show any variance in the data, the number of samples was increased to six per arm, thereby increasing statistical power.

## Results: (see supplemental digital content)

A detailed description of ventilatory settings can be found in the supplement along with a summary of the total amounts of all drugs used during the experiments, and arterial and venous blood work results (Supplementary Tables S1–S11) (Bassi et al., 2022a).

Eighteen female pigs were studied, with similar weights. (Bassi et al., 2022a). The median weights were 64 kg (62–65) in the MV group (n = 6), 67 kg (64–84) in the MV + PNS50% group (n = 6), and 69 kg (67–73) in the MV + PNS100% group (n = 6) (Supplementary Tables S1) (Bassi et al., 2022a). Physiological parameters, mean arterial pressure, central venous pressure, temperature, heart rate, glucose levels, PaCO_2_, and fluid balance (input minus output) were within the normal range (Bassi et al., 2022a) for all animals from all groups (Supplementary Tables S2–S14). (Bassi et al., 2022a). Bilateral phrenic-nerve capture was achieved in all animals receiving PNS. (Bassi et al., 2022a).

In addition, We did not identify a statistically significant difference between the total administered doses, normalized to weight, of propofol, midazolam, fentanyl, ketamine, phenylephrine, norepinephrine bitartrate, and oleic acid between the groups (Supplementary Table S11); (Bassi et al., 2022a).

### Systemic inflammatory markers in the serum: (supplemental digital content)

Serum concentration levels of systemic inflammatory markers IL-1α, IL-1β, IL-6, IL-8, IL-10, and TNFα were not significantly different between the groups (see Supplementary Table S12). However, IFN-γ and GM-CSF serum concentrations were significantly different between the groups. (Bassi et al., 2022a). The MV + PNS50% group showed significantly lower IFN-γ serum concentration at study end compared to the MV + PNS100% group, *p* = 0.0133. The MV + PNS100% group showed significantly higher GM-CSF serum concentration at study end compared to the MV group, *p* = 0.0475 (Bassi et al., 2022a).

### Hippocampal results (Bassi et al., 2022a):

Differences in the apoptotic cell, microglia, and reactive astrocyte percentages were statistically significant between the groups, respectively *p* = 0.0002, *p* = 0.0002, and *p* = 0.0002 (Supplementary Table S15); (Bassi et al., 2022a).

**FIGURE 1:**
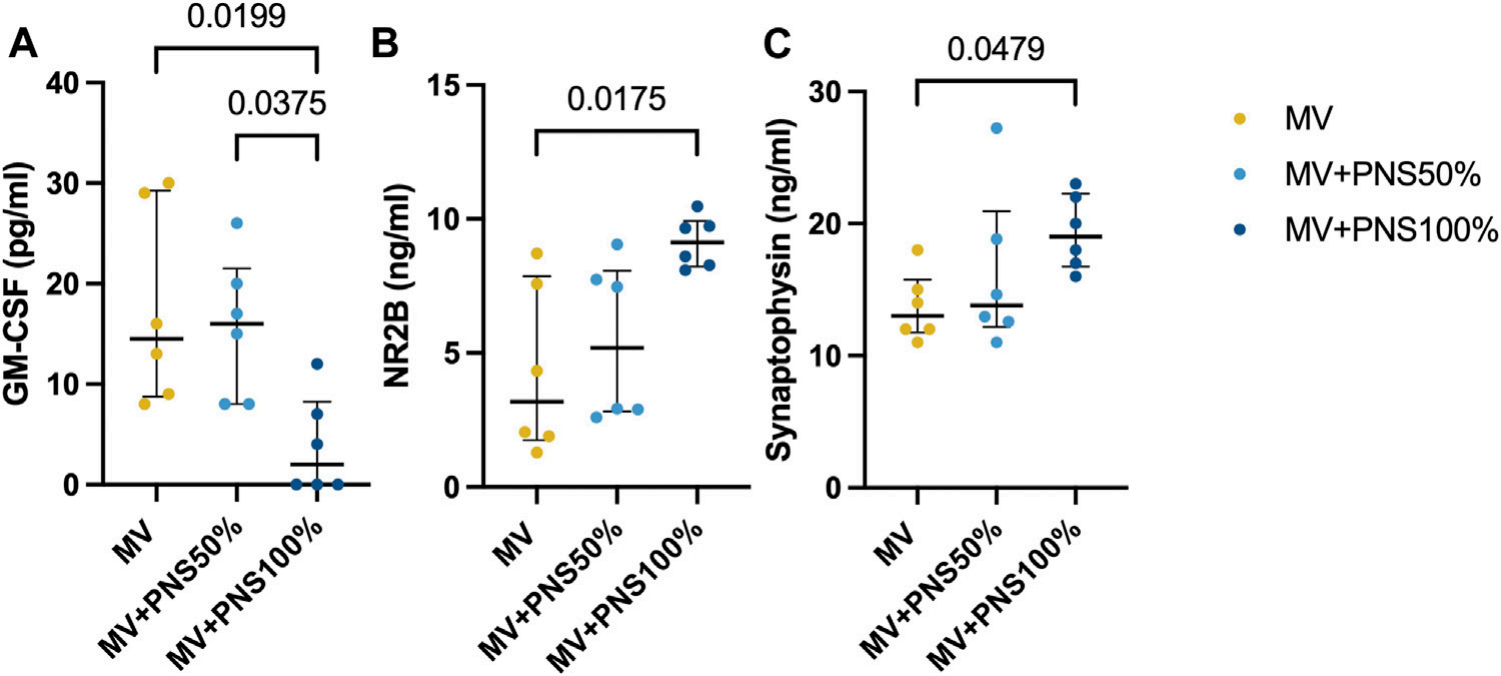
Dot plots show significant differences in hippocampal tissue concentrations between the groups. Data are expressed as median and interquartile range. **(A)** Dot plots show a significant difference in hippocampal tissue concentration of granulocyte macrophage-colony stimulating factor (GM-CSF) between the MV + PNS50% group, 15.0 pg/ml (9.8–20.3), and the MV + PNS100% group, 0.0 pg/ml (0.0–8.4), *p* = 0.0375, and a significant difference between the MV group, 15.0 pg/ml (8.8–28.1), and the MV + PNS100% group, 0.0 pg/ml (0.0–8.4), *p* = 0.0199. **(B)** Dot plots show a significant difference in hippocampal tissue concentration of N-methyl-D-aspartate receptor 2B (NR2B) between the MV group, 3.0 ng/ml (2.0–8.0), and the MV + PNS100% group, 9.0 ng/ml (8.0–10.0), *p* = 0.0175. **(C)** Dot plots show a significant difference in hippocampal tissue concentration of synaptophysin between the MV group, 13.0 ng/ml (12.0–16.0), and the MV + PNS100% group, 19.0 ng/ml (17.0–23.0), *p* = 0.0479.

Hippocampal inflammatory tissue concentrations are reported in Table 1. All inflammatory markers analyzed in the hippocampal tissue showed no statistically significant differences between groups, except for GM-CSF, *p* < 0.0001 (Figure 1A). The MV + PNS100% group showed the lowest GM-CSF hippocampal tissue concentration among the groups.

**TABLE 1:** Table 1 shows the results of tissue markers concentrations in the hippocampus and brainstem.

Tissue markers	Hippocampus			Brainstem		
Median (IQ)	MV	MV + PNS50%	MV + PNS100%	MV	MV + PNS50%	MV + PNS100%
IL-1α (pg/ml)	0.7 (0.6–0.8)	0.8 (0.3–1.4)	1.0 (0.8–1.2)	0.8 (0.7–1.3)	0.8 (0.6–0.9)	0.7 (0.4–1.3)
IL-1β (pg/ml)	0.0 (0.0–0.0)	0.1 (0.0–5.1)	0.0 (0.0–3.0)	10.4 (6.1–29.9)	8.9 (5.1–21.8)	3.9 (0.0–6.0)
IL-6 (pg/ml)	0.0 (0.0–3.0)	0.0 (0.0–2.0)	0.0 (0.0–2.0)	0.0 (0.0–3.6)	0.0 (0.0–1.8)	0.0 (0.0–2.8)
IL-8 (pg/ml)	27.0 (10.0–58.8)	42.4 (10.0–76.0)	27.0 (15.0–30.2)	36.2 (29.1–45.1)	29.3 (16.3–63.0)	0.0 (0.0–16.3)
IL-10 (pg/ml)	0.0 (0.0–0.0)	0.0 (0.0–0.0)	0.0 (0.0–0.0)	0.0 (0.0–0.0)	0.0 (0.0–0.0)	0.0 (0.0–0.0)
TNFα (pg/ml)	0.0 (0.0–0.0)	0.0 (0.0–0.0)	0.0 (0.0–0.0)	0.0 (0.0–0.0)	0.0 (0.0–0.0)	0.0 (0.0–0.0)
GM-CSF (pg/ml)	14.5 (8.8–28.1)	16.0 (9.8–20.3)	2.0 (0.0–8.4)	0.0 (0.0–13.8)	0.0 (0.0–10.5)	0.0 (0.0–0.0)
IFN-γ (pg/ml)	66.5 (56.0–101.2)	99.0 (78.5–117.6)	99.8 (66.0–99.7)	38.2 (25.4–72.2)	58.8 (42.2–79.8)	93.2 (79.8–130.3)
Synaptophysin (ng/ml)	13.0 (12.0–16.0)	14.0 (12.6–21.0)	19.0 (17.0–23.0)	12.0 (10.0–17.5)	14.5 (11.4–16.7)	13.3 (11.5–14.7)
Synapsin (ng/ml)	1.6 (1.5–1.7)	1.7 (1.6–1.9)	1.5 (1.4–1.8)	2.1 (1.8–2.4)	2.4 (1.9–2.7)	2.1 (1.9–2.1)
NR2A (ng/ml)	5.0 (4.0–8.0)	5.0 (5.0–8.0)	7.0 (6.0–9.0)	3.7 (3.4–5.0)	4.4 (4.2–8.1)	7.2 (6.3–8.2)
NR2B (ng/ml)	3.2 (2.0–7.7)	5.2 (3.0–8.0)	9.1 (8.2–10.1)	11.0 (3.1–12.0)	10.5 (2.9–14.0)	12.8 (11.3–15.6)
DLG4 (ng/ml)	3.1 (2.8–3.4)	3.1 (2.6–3.3)	3.0 (2.9–3.7)	3.3 (3.0–4.4)	3.0 (2.7–3.4)	3.0 (2.8–3.2)

Concentrations of hippocampal synaptic markers are reported in Table 1. Statistically significant differences were found between the MV + PNS100% and the MV groups for NR2B and synaptophysin (SYP) tissue concentrations, respectively *p* = 0.0175, and *p* = 0.0479 (Figures 1B, C).

### Brainstem results

**FIGURE 2:**
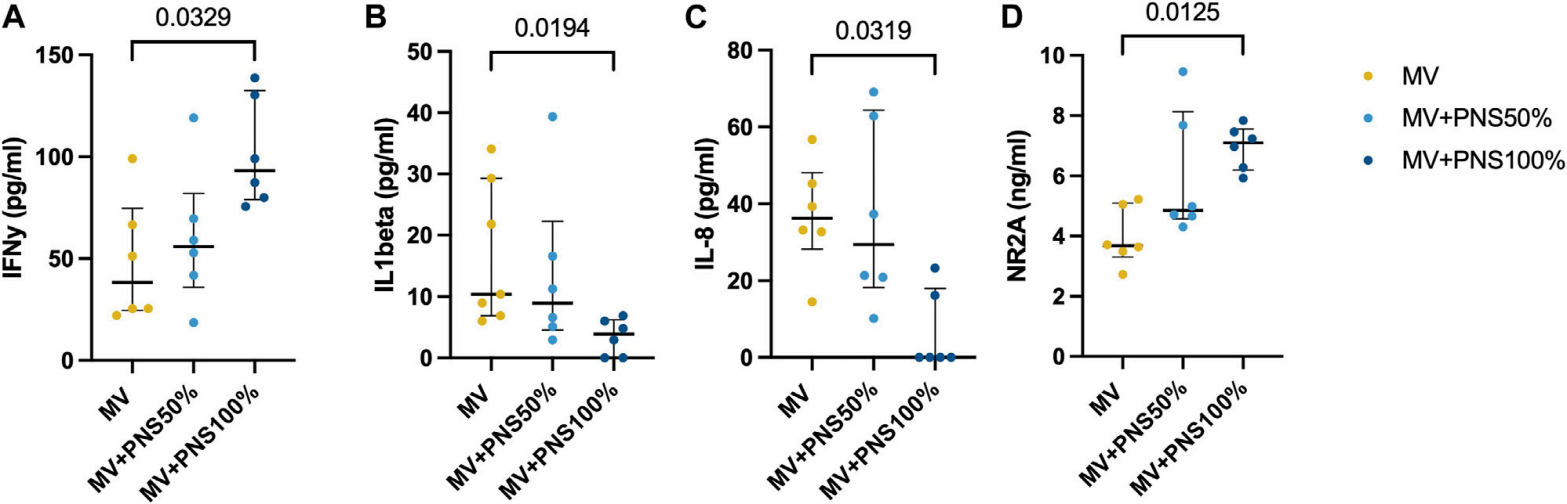
Dot plots show significant differences in brainstem tissue concentrations between the groups. Data are expressed as median and interquartile range. **(A)** Dot plots show a significant difference in brainstem tissue concentration of interferon-gamma (IFNγ) between the MV group, 45.0 pg/ml (22.0–82.2), and the MV + PNS100% group, 84.4 pg/ml (74.0–116.6), *p* = 0.0329. **(B)** Dot plots show a significant difference in brainstem tissue concentration of interleukin-1β (IL-1β) between the MV group, 10.0 pg/ml (7.0–22.0), and the MV + PNS100% group, 4.2 pg/ml (0.0–7.0), *p* = 0.0194. **(C)** Dot plots show a significant difference in brainstem tissue concentration of interleukin 8 (IL-8) between the MV group, 36.2 pg/ml (28.2–48.1), and the MV + PNS100% group, 0.0 pg/ml (0.0–18.0), *p* = 0.0319. **(D)** Dot plots show a significant difference in brainstem tissue concentration of N-methyl-D-aspartate receptor 2A (NR2A) between the MV group, 4.0 ng/ml (3.0–5.0), and the MV + PNS100% group, 7.0 ng/ml (6.0–8.0), *p* = 0.0125.

Brainstem inflammatory tissue concentrations are reported in Table 1. Differences in IFN-γ, IL-1β, and IL-8 brainstem tissue concentrations were statistically significant between the MV + PNS100% and the MV groups, respectively *p* = 0.0017, *p* = 0.0039, and *p* < 0.0001 (Figures 2A–C).

Concentrations of brainstem synaptic markers are reported in Table 1. Statistically significant differences between the MV + PNS100% and the MV groups were found for NR2A brainstem tissue concentration, *p* = 0.0125 (Figure 2D).

### Correlation between hippocampal apoptosis and microglia percentages and GM-CSF tissue concentration

**FIGURE 3:**
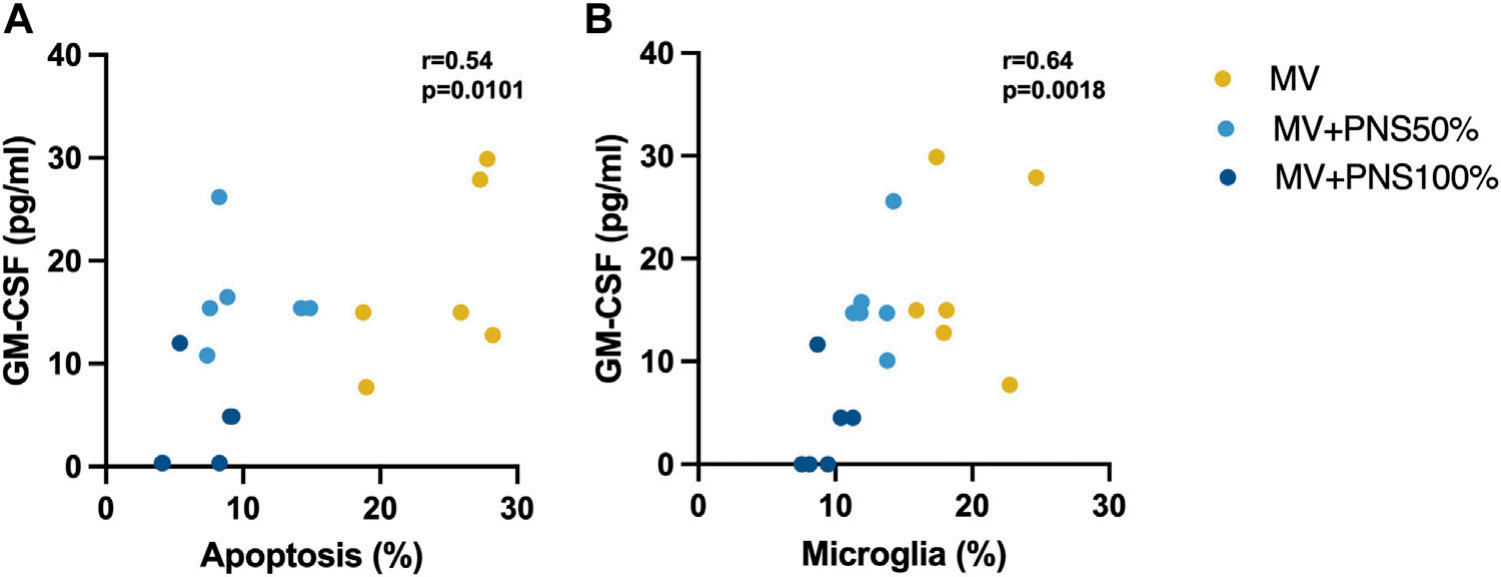
**(A)** Scatter plot shows a significant positive, linear, and moderate correlation between hippocampal apoptosis percentages and granulocyte macrophage-colony stimulating factor (GM-CSF) hippocampal tissue concentrations, r = 0.54, *p* = 0.0101. **(B)** Scatter plot shows a significant positive, linear, and moderate correlation between hippocampal microglia percentages and GM-CSF hippocampal tissue concentrations, r = 0.64, *p* = 0.0018.

Positive, linear, and moderate correlations were found between hippocampal apoptosis percentages and GM-CSF hippocampal tissue concentrations, r = 0.54, *p* = 0.0101 (Figure 3A), and between hippocampal microglia percentages and GM-CSF hippocampal tissue concentrations, r = 0.64, *p* = 0.0018 (Figure 3B).

### Correlation between hippocampal apoptosis, microglia and astrocyte percentages and synaptic marker tissue concentration

**FIGURE 4:**
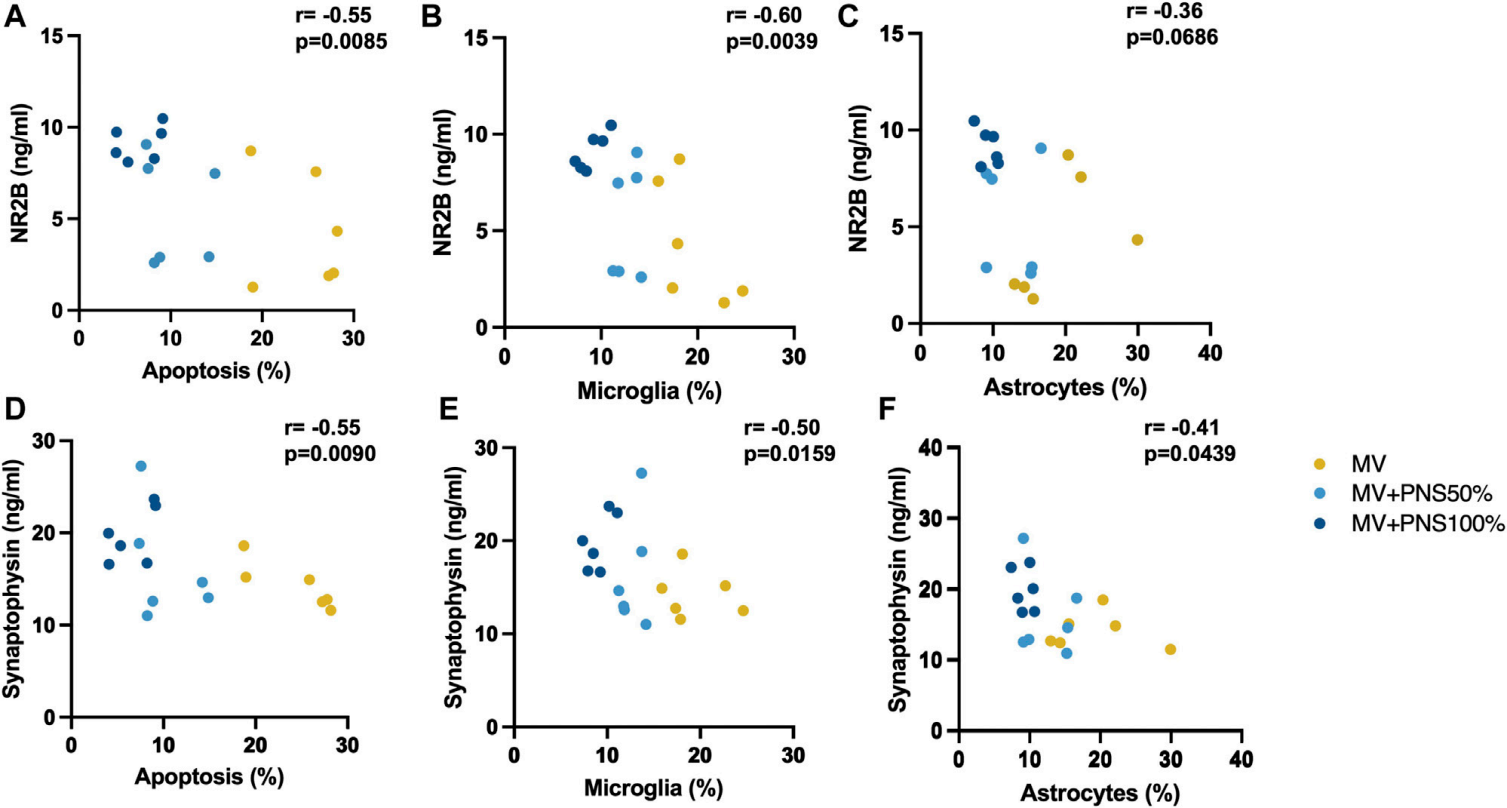
**(A)** Scatter plot shows a significant negative, linear, and moderate correlation between hippocampal apoptosis percentages and N-methyl-D-aspartate receptor 2B (NR2B) hippocampal tissue concentrations, r = − 0.55, *p* = 0.0085. **(B)** Scatter plot shows a significant correlation between microglia percentages and NR2B hippocampal tissue concentrations, r = − 0.60, *p* = 0.0039. **(C)** Scatter plot shows a negative, linear, and moderate correlation tending to significance between hippocampal astrocyte percentages and NR2B hippocampal tissue concentrations, r = − 0.36, *p* = 0.0686. **(D)** Scatter plot shows a significant negative, linear, and moderate correlation between hippocampal apoptosis percentages and synaptophysin hippocampal tissue concentrations, r = − 0.55, *p* = 0.0090. **(E)** Scatter plot shows a significant negative, linear, and moderate correlation between hippocampal microglia percentages and SYP hippocampal tissue concentrations, r = − 0.50, *p* = 0.0159. **(F)** Scatter plot shows a significant negative, linear, and moderate correlation between hippocampal astrocyte percentages and synaptophysin hippocampal tissue concentrations, r = −0.41, *p* = 0.0439.

Significant negative, linear, and moderate correlations were found between hippocampal apoptosis percentages and NR2B hippocampal tissue concentrations, r = − 0.55, *p* = 0.0085 (Figure 4A), and between microglia percentages and NR2B hippocampal tissue concentrations, r = − 0.60, *p* = 0.0039 (Figure 4B), and a negative, linear, and moderate correlation tending to significance between hippocampal astrocyte percentages and NR2B hippocampal tissue concentrations, r = − 0.36, *p* = 0.0686 (Figure 4C).

Significant negative, linear, and moderate correlations were found between hippocampal apoptosis percentages and SYP hippocampal tissue concentrations, r = − 0.55, *p* = 0.0090 (Figure 4D), hippocampal microglia percentages and SYP hippocampal tissue concentrations, r = − 0.50, *p* = 0.0159 (Figure 4E), and hippocampal astrocyte percentages and SYP hippocampal tissue concentrations, r = −0.41, *p* = 0.0439 (Figure 4F).

## Discussion

In this moderate-ARDS preclinical study, we found that hippocampal apoptosis and inflammation were associated with lower concentrations of two synaptic markers in the hippocampus, namely NR2B and synaptophysin. The brainstem showed greater tissue concentrations of two pro-inflammatory markers, IL-1β and IL-8, in the group that received only lung-protective MV in association with moderate ARDS, and phrenic nerve stimulation on every breath resulted in significantly lower tissue concentrations of those markers. These results complement our previous findings, which showed that moderate ARDS and MV resulted in greater hippocampal apoptosis and neuroinflammation, by showing that moderate ARDS and MV are also associated with greater tissue concentration of inflammatory markers in the brainstem and lower concentration of synaptic markers in the hippocampus. (Bassi et al., 2021c).

Previously, we published that the group with moderate ARDS undergoing MV for 12 h had greater hippocampal apoptosis and inflammation, compared to the group that received PNS on every breath (Bassi et al., 2022a). This study complements these findings by showing that the group with moderate ARDS undergoing MV for 12 h also had greater hippocampal tissue concentrations of GM-CSF, a proinflammatory marker, compared to the group that received PNS on every breath (Bassi et al., 2022a). The correlation between hippocampal microglia percentages and hippocampal tissue concentrations of GM-CSF supports the observation that ARDS and MV are associated with neuroinflammation. The group receiving PNS on every breath also had greater hippocampal tissue concentrations of N-methyl-D-aspartate receptor 2B compared to the other groups. A low concentration of NR2B has been associated with cognitive impairment (Rao et al., 2012). N-methyl-D-aspartate receptors are also important for cell survival and death (Martel et al., 2009). Preclinical studies have shown an association between lower concentrations of synaptic N-methyl-d-aspartate receptors and greater cell apoptosis by the activation of the intrinsic apoptotic cascade (Martel et al., 2009). NR2 receptors seem to assist in the regulation of the gene expression of cyclic AMP response element binding protein, also called CREB (Martel et al., 2009). This gene modulates the calcium neuronal influx depending on the location in which the NR2 was activated. Synaptic NR2 receptors when activated and in greater concentration are neuronally protective and in lower concentration and activation induce cellular apoptosis by triggering mitochondrial death. On the other hand, greater activation of extrasynaptic NR2 receptors triggers cellular apoptosis and lower concentration of those are neuroprotective (Martel et al., 2009). During normal synaptic activity, the synaptic NR2 receptors are stimulated and activated first and if the concentration of NR2 passes the physiological threshold it stimulates and activates extrasynaptic NR2 receptors, triggering apoptosis which results in a reduction of synaptic NR2 receptors (Martel et al., 2009). Thus, the greater the apoptosis percentage is the lower the concentration of NR2 synaptic receptors should be. Our results showed a negative correlation between hippocampal apoptosis percentages and the hippocampal tissue concentration of NR2B, which argues in favor of an association between NR2B concentration and its effect on cell survival (Bassi et al., 2022a). This is an important finding because hippocampal inflammation and cellular apoptosis have been linked to cognitive dysfunction (Jalleh et al., 2012; Tannenholz et al., 2014). For instance, a preclinical study showed that mice ventilated for 6 hours had greater levels of hippocampal microglia and worse cognitive scores compared to mice ventilated for 1 hour (Chen et al., 2015).

Although our study did not analyze cognitive function after extubation, hippocampal inflammation and low concentrations of NR2B are both associated with cognitive impairment (Martel et al., 2009; Janz et al., 2010; van Munster et al., 2011). In addition, synaptophysin is a synaptic marker whose concentration is strongly and positively associated with cognitive function (Martel et al., 2009; Rao et al., 2012). For instance, clinical and preclinical studies have shown that lower synaptophysin concentration is associated with lower cognitive scores, and a low concentration of synaptophysin is observed in patients with dementia, compared to patients without dementia (Martel et al., 2009; Rao et al., 2012). Our study showed that PNS on every breath resulted in the highest synaptophysin hippocampal tissue concentration among the groups. In our ARDS model, the mitigation of cellular apoptosis and inflammation observed with PNS was considerable, compared to the group that received only MV. A moderate, negative, linear correlation between hippocampal apoptosis percentages and synaptophysin hippocampal tissue concentrations, between hippocampal microglia percentages and synaptophysin hippocampal tissue concentrations, and between hippocampal astrocyte percentages and synaptophysin hippocampal tissue concentrations support the observation that PNS on every breath resulted in neuroprotection. These findings align with the current literature that shows neuroinflammation and cellular apoptosis are linked to low concentrations of N-methyl-d-aspartate receptors and synaptic markers (Martel et al., 2009).

In previously published work, our group reported that the serum concentrations of GM-CSF at study end were significantly different between the group that received PNS on every breath and the MV group (Bassi et al., 2022a). The group receiving PNS every breath showed a significantly higher serum concentration of GM-CSF, compared to the MV group. GM-CSF is a protein that can cross the blood-brain barrier, regardless of blood-brain barrier permeability (Daniels and Klein, 2015; Kiyota et al., 2018); therefore, it might reasonably be expected that the hippocampal tissue concentrations of GM-CSF would be higher in the MV + PNS100% group as well. However, the lowest hippocampal tissue concentrations of GM-CSF combined with the highest GM-CSF serum concentrations at study end were observed in the MV + PNS100% group. This may help to explain the mechanism of action for the neuroprotection observed with PNS. It has been hypothesized that PNS could promote neuroprotection by either assisting in reducing systemic inflammation or by modulating the neural signaling to the hippocampus via the vagus nerve (Bassi et al., 2021b; Bassi et al., 2022a). If PNS promotes neuroprotection by reducing systemic inflammation, it would be expected to find comparable behaviors between the concentrations of GM-CSF in the serum and in the hippocampal tissue, which was not the case. Thus, these findings support the argument that PNS modulates neural signaling, resulting in a different behavior between the concentrations of GM-CSF in the serum and those in the tissue (Bassi et al., 2021b; Bassi et al., 2022a).

The brainstem is a vital structure that regulates breathing (Ikeda et al., 2017; Sheikhbahaei et al., 2018). Our study found that the MV group had the highest brainstem tissue concentration of two pro-inflammatory markers, IL-1β and IL-8. In contrast, the MV + PNS100% group had the lowest brainstem tissue concentration of these two markers. Thus, the neuroprotection provided by PNS was not restricted to the hippocampus, but also extended to the brainstem. These findings have clinical importance because hippocampus and brainstem inflammation have been linked, respectively, to cognitive dysfunction and difficulty to wean off mechanical ventilation (Janz et al., 2010; van Munster et al., 2011; Sheikhbahaei et al., 2018). A post-mortem study in ARDS patients who had delirium prior to death showed greater hippocampal inflammation compared to ARDS patients who had not delirium prior to death (Janz et al., 2010). A preclinical study showed that inducing brainstem inflammation resulted in lower breathing variability and lower breathing adaptability to exercise (Sheikhbahaei et al., 2018). Clinical studies have shown that lower breathing variability is associated with greater in-hospital mortality and difficulty to wean-off MV (van den Bosch et al., 2021; Garrido et al., 2018). Interestingly, the tissue and serum concentrations of IFN-γ were both highest in the group receiving PNS on every breath. In porcine models, IFN-γ regulates and controls inflammation (Daniels and Klein, 2015; Ferrara et al., 2022). Higher serum concentration of IFN-γ has also been shown to decrease blood-brain barrier permeability, resulting in neuroprotection by “closing” the blood-brain barrier (Daniels and Klein, 2015).

Our study had limitations. Although we analyzed the concentration of N-methyl-d-aspartate receptors, NR2A and NR2B, in the hippocampus and brainstem, our study was not able to investigate the origin of these receptors, synaptic or extra-synaptic. This would have been valuable, because greater concentrations of extra-synaptic NR2 receptors are associated with greater cellular apoptosis (Martel et al., 2009). Despite this limitation, it is likely that the greater concentration of NR2 receptors observed in this study in the MV + PNS100% group was synaptic, due to the lower cellular apoptosis observed and previously reported in this group. The systemic inflammatory markers were analyzed directly from serum samples, which could confound our results. Previously, it has been demonstrated that TNFα, a pro-inflammatory marker, is stored in intracellular vesicles in the leucocytes before its release (Bernardino et al., 2008). During the process of serum analysis, the TNFα serum concentration may have been affected by the centrifugation process, resulting in a greater concentration in the serum samples in all groups, and therefore confounding our results. While cognitive function was not assessed in our study, lower concentrations of synaptic markers have been linked to cognitive impairment in preclinical and clinical studies, (Sze Chun et al., 1997), (Kiyota et al., 2018) Our study only investigated the effect of PNS in female pigs. Our study was not designed to investigate the mechanism of action for these findings. It is unknown whether the findings were a result of a diaphragm contraction, direct stimulation of the phrenic afferent fibres or secondary to another cause. This is because, in a porcine ARDS model, it has been shown that computer-controlled ventilation set to result in a greater breathing variability within the respiratory cycles resulted in better lung physiology and gas exchange compared to the control group (Lefevre et al., 1996; Rocha et al., 2022). Thus, it is unknown whether the neuroprotective effect of phrenic nerve stimulation is a result of a greater breathing variability within the respiratory cycles which could reduce lung collapse, and/or improved lung compliance, which in turn would stimulate more pulmonary stretch receptors increasing the vagus nerve signal, and therefore leading to neuroprotection. Future studies should be designed to investigate the possible mechanisms of action for the neuroprotection resulting from phrenic nerve stimulation. Although the hybrid ventilatory strategy used in this study mitigated hippocampal and brainstem inflammation in deeply sedated animals in ICU conditions, clinical studies are needed to investigate the clinical outcomes of this strategy.

## Conclusion

Our study showed that pigs with moderate ARDS undergoing lung-protective mechanical ventilation for 12 h exhibited the lowest hippocampal concentrations of two synaptic markers, NR2B and synaptophysin, and the highest brainstem tissue concentrations of IL-1β and IL-8. Moderate-ARDS pigs undergoing lung-protective mechanical ventilation for 12 h in conjunction with phrenic nerve stimulation on every breath exhibited the highest hippocampal concentrations of NR2B and synaptophysin, and the lowest brainstem tissue concentrations of IL-1β and IL-8. Future studies should investigate the mechanism of neuroprotective effect resulting from phrenic nerve stimulation on every breath.

## Data Availability

The raw data supporting the conclusion of this article will be made available by the authors, without undue reservation.
