# Supplementary material for: Phrenic nerve stimulation mitigates hippocampal and brainstem inflammation in an ARDS model
Source: Front Physiol. 2023 May 5;14:1182505. doi: 10.3389/fphys.2023.1182505 (PMC10196250; doi:10.3389/fphys.2023.1182505)
Supplement: Supplementary file 1 [file DataSheet1.docx]

Figure S1. Example waveforms from an MV+PNS50% subject showing: volume (blue line), airflow (green line), and pressure (red line) for a ventilator-delivered breath without phrenic-nerve stimulation (A), and for a ventilator-delivered breath with phrenic-nerve stimulation (B). A reduction of pressure is observed on the ventilator-delivered breath with phrenic-nerve stimulation (B), with no change in either volume or airflow, compared to the ventilator-delivered breath without phrenic-nerve stimulation (A).

**Results:**

All subjects were between 4 and 5 months old. Subjects’ weights before the start of the experiment are shown in Table S1. No statistically significant difference was found between the weights of the mechanically ventilated groups.

Table S1. Subjects’ weights before the start of the experiment.

| **MV**  **Group** | | **MV+PNS50% Group** | | **MV+PNS100% Group** | |
| --- | --- | --- | --- | --- | --- |
| Subject | Weight (kg) | Subject | Weight (kg) | Subject | Weight (kg) |
| 1 | 67 | 1 | 65 | 1 | 67 |
| 2 | 63 | 2 | 83 | 2 | 63 |
| 3 | 62 | 3 | 87 | 3 | 67 |
| 4 | 61 | 4 | 66 | 4 | 71 |
| 5 | 64 | 5 | 62 | 5 | 77 |
| 6 | 64 | 6 | 68 | 6 | 71 |
| Median | 63 | Median | 67 | Median | 69 |

All subjects from all groups had body temperature in a normal range for the duration of the study. Mean arterial pressure and central venous pressure are shown in Table S2. Hemodynamic stability was achieved after lung injury in all subjects. The tidal volumes delivered for all subjects in the MV, MV+PNS50%, and MV+PNS100% groups were constant at 8 ml/kg, in accordance with lung-protective mechanical ventilation recommendations.^1^

| **Mean arterial pressure and central venous pressure** | **Median**  **(IQR)** | | | **p-value (Kruskal-Wallis test)** | **p-value**  **(Dunn’s multiple comparison test)** | |
| --- | --- | --- | --- | --- | --- | --- |
|  | **MV**  **group**  **(n=6)** | **MV+PNS50% group**  **(n=6)** | **MV+PNS100% group**  **(n=6)** |  |  |  |
| Mean arterial pressure, study start (mmHg) | 66  (61-69) | 64  (56-76) | 70  (61-82) | 0.6148 | MV vs. MV+PNS50% | --- |
|  |  |  |  |  | MV vs. MV+PNS100% | --- |
|  |  |  |  |  | MV+PNS50% vs. MV+PNS100% | --- |
| Mean arterial pressure, lung injury achieved (mmHg) | 88  (83-92) | 79  (75-95) | 84  (76-94) | 0.6676 | MV vs. MV+PNS50% | --- |
|  |  |  |  |  | MV vs. MV+PNS100% | --- |
|  |  |  |  |  | MV+PNS50% vs. MV+PNS100% | --- |
| Mean arterial pressure, study end (mmHg) | 73  (65-74) | 73  (66-82) | 85  (82-90) | 0.0057 | MV vs. MV+PNS50% | ns |
|  |  |  |  |  | MV vs. MV+PNS100% | 0.0124 |
|  |  |  |  |  | MV+PNS50% vs. MV+PNS100% | ns |
| Central venous pressure, study start (cmH_2_O) | 7  (6-9) | 9  (8-10) | 7  (5-8) | 0.2034 | MV vs. MV+PNS50% | --- |
|  |  |  |  |  | MV vs. MV+PNS100% | --- |
|  |  |  |  |  | MV+PNS50% vs. MV+PNS100% | --- |
| Central venous pressure, lung injury achieved (cmH_2_O) | 9  (8-10) | 10  (7-12) | 8  (7-9) | 0.5324 | MV vs. MV+PNS50% | --- |
|  |  |  |  |  | MV vs. MV+PNS100% | --- |
|  |  |  |  |  | MV+PNS50% vs. MV+PNS100% | --- |
| Central venous pressure, study end (cmH_2_O) | 7  (6-9) | 8  (5-9) | 5  (3-6) | 0.0402 | MV vs. MV+PNS50% | ns |
|  |  |  |  |  | MV vs. MV+PNS100% | ns |
|  |  |  |  |  | MV+PNS50% vs. MV+PNS100% | ns |

Table S2. Mean arterial pressure (mmHg) and central venous pressure (cmH_2_O) for all mechanically ventilated groups at three different time-points, study start, lung-injury achieved, and study end.

**Ventilator Settings:**

Ventilator settings for all subjects in the MV, MV+PNS50%, and MV+PNS100% groups are shown in Table S3, Table S9, and Table S10. Differences in respiratory rate between the PNS groups were statistically significant. Differences in all the other variables were not statistically significant between the groups.

Table S3. Ventilator settings for all mechanically ventilated groups. The values reported are the mean values over the duration of the study.

| **Ventilator settings** | **MV group**  **Median**  **(IQR)** | **MV+PNS50% group**  **Median**  **(IQR)** | **MV+PNS100% group**  **Median**  **(IQR)** | **Kruskal-Wallis test**  **(p-value)** | **Dunn’s multiple comparison**  **Test**  **(p-value)** | |
| --- | --- | --- | --- | --- | --- | --- |
| **Respiratory rate** | 20  (20-21) | 20  (20-21) | 21  (21-22) | 0.0189 | LI-MV vs. LI-MV+TTDN50% | ns |
|  |  |  |  |  | LI-MV vs. LI-MV+TTDN100% | ns |
|  |  |  |  |  | LI-MV+TTDN50% vs. LI-MV+TTDN100% | 0.0207 |
| **Tidal volume (ml/kg)** | 8  (8-8) | 8  (8-8) | 8  (8-8) | ns | LI-MV vs. LI-MV+TTDN50% | --- |
|  |  |  |  |  | LI-MV vs. LI-MV+TTDN100% | --- |
|  |  |  |  |  | LI-MV+TTDN50% vs. LI-MV+TTDN100% | --- |
| **FiO_2_** | 0.40  (0.37-0.42) | 0.33  (0.33-0.36) | 0.32  (0.32-0.47) | 0.0274 | LI-MV vs. LI-MV+TTDN50% | ns |
|  |  |  |  |  | LI-MV vs. LI-MV+TTDN100% | ns |
|  |  |  |  |  | LI-MV+TTDN50% vs. LI-MV+TTDN100% | ns |

**Fluid Balance:**

Fluid balance for subjects in the MV, MV+PNS50%, and MV+PNS100% groups was closely monitored throughout the experiment. Positive fluid balances were observed in the range of 1.2 ml/kg/hr to 1.6 ml/kg/hr. No statistically significant difference was found between the groups (Table S4).

Table S4. Fluid balance (fluid intake minus fluid loss) for all groups.

| **MV**  **Group** | | **MV+PNS50% Group** | | **MV+PNS100% Group** | |
| --- | --- | --- | --- | --- | --- |
| Subject | Fluid balance  (ml/kg/hr) | Subject | Fluid balance  (ml/kg/hr) | Subject | Fluid balance  (ml/kg/hr) |
| 1 | 1.2 | 1 | 1.4 | 1 | 1.3 |
| 2 | 1.4 | 2 | 1.3 | 2 | 1.2 |
| 3 | 1.2 | 3 | 1.4 | 3 | 1.2 |
| 4 | 1.3 | 4 | 1.2 | 4 | 1.2 |
| 5 | 1.2 | 5 | 1.4 | 5 | 1.2 |
| 6 | 1.3 | 6 | 1.6 | 6 | 1.3 |
| Median | 1.4 | Median | 1.3 | Median | 1.2 |
| Kruskal-Wallis test | | | | | |
| ns | | | | | |

**Blood Gas Samples:**

All mechanically ventilated subjects had arterial blood samples taken, to analyze PaO_2_, PaCO_2_, and pH. The mechanically ventilated subjects had arterial samples taken at baseline, every hour, at the end of the study or when needed. Tables S5 - S10 show the results for the mechanically ventilated groups.

Table S5. Arterial blood gas results before the induction of lung injury for the MV, MV+TTDN50%, and MV+TTDN100% groups (medians and IQR for all samples taken during the experiment).

| **Arterial blood gas measurements** | **MV group**  **Median**  **(IQR)** | **MV+PNS50% group**  **Median**  **(IQR)** | **MV+PNS100% group**  **Median**  **(IQR)** | **Kruskal-Wallis test**  **(p-value)** |
| --- | --- | --- | --- | --- |
| **PaO_2_**  **(mmHg)** | 141  (92-152) | 128  (110-152) | 121  (111-147) | ns |
| **PaCO_2_**  **(mmHg)** | 52  (47-54) | 50  (47-55) | 48  (46-54) | ns |
| **pH** | 7.44  (7.42-7.47) | 7.44  (7.39-7.45) | 7.44  (7.38-7.46) | ns |

Table S6. PaO_2_ for all mechanically ventilated subjects at study start, after lung injury was achieved, and at study end.

| **PaO_2_**  **(mmHg)** | | | | | | | | |
| --- | --- | --- | --- | --- | --- | --- | --- | --- |
| **Study Start** | | | **Lung Injury Achieved** | | | **Study End** | | |
| **MV**  **group** | **MV+TTDN50%**  **group** | **MV+TTDN100%**  **group** | **MV**  **group** | **MV+TTDN50%**  **group** | **MV+TTDN100%**  **group** | **MV**  **group** | **MV+TTDN50%**  **group** | **MV+TTDN100%**  **group** |
| 183 | 124 | 137 | 56 | 52 | 66 | 92 | 128 | 109 |
| 271 | 136 | 159 | 47 | 45 | 57 | 184 | 132 | 124 |
| 297 | 142 | 158 | 58 | 60 | 59 | 158 | 99 | 117 |
| 153 | 167 | 161 | 67 | 61 | 58 | 133 | 125 | 144 |
| 154 | 116 | 147 | 61 | 50 | 56 | 124 | 164 | 135 |
| 133 | 118 | 154 | 59 | 65 | 59 | 144 | 156 | 156 |

Table S7. PaCO_2_ for all mechanically ventilated subjects at study start, after lung injury was achieved, and at study end.

| **PaCO_2_**  **(mmHg)** | | | | | | | | |
| --- | --- | --- | --- | --- | --- | --- | --- | --- |
| **Study Start** | | | **Lung Injury Achieved** | | | **Study End** | | |
| **MV**  **group** | **MV+TTDN50%**  **group** | **MV+TTDN100%**  **group** | **MV**  **group** | **MV+TTDN50%**  **group** | **MV+TTDN100%**  **group** | **MV**  **group** | **MV+TTDN50%**  **group** | **MV+TTDN100%**  **group** |
| 55 | 65 | 61 | 47 | 55 | 52 | 46 | 46 | 52 |
| 53 | 50 | 60 | 54 | 63 | 57 | 41 | 53 | 44 |
| 57 | 49 | 58 | 56 | 51 | 62 | 51 | 42 | 46 |
| 63 | 43 | 56 | 49 | 41 | 50 | 47 | 35 | 42 |
| 56 | 60 | 50 | 53 | 51 | 49 | 48 | 57 | 43 |
| 66 | 67 | 55 | 50 | 64 | 58 | 51 | 50 | 40 |

Table S8. pH for all mechanically ventilated subjects at study start, after lung injury was achieved, and at study end.

| **pH** | | | | | | | | |
| --- | --- | --- | --- | --- | --- | --- | --- | --- |
| **Study Start** | | | **Lung Injury Achieved** | | | **Study End** | | |
| **MV**  **group** | **MV+TTDN50%**  **group** | **MV+TTDN100%**  **group** | **MV**  **group** | **MV+TTDN50%**  **group** | **MV+TTDN100%**  **group** | **MV**  **group** | **MV+TTDN50%**  **group** | **MV+TTDN100%**  **group** |
| 7.41 | 7.48 | 7.39 | 7.38 | 7.33 | 7.32 | 7.40 | 7.50 | 7.47 |
| 7.51 | 7.43 | 7.43 | 7.36 | 7.40 | 7.34 | 7.48 | 7.42 | 7.52 |
| 7.46 | 7.45 | 7.43 | 7.34 | 7.44 | 7.35 | 7.46 | 7.47 | 7.44 |
| 7.43 | 7.46 | 7.45 | 7.38 | 7.43 | 7.33 | 7.47 | 7.52 | 7.48 |
| 7.42 | 7.36 | 7.41 | 7.36 | 7.32 | 7.40 | 7.49 | 7.39 | 7.44 |
| 7.40 | 7.40 | 7.44 | 7.28 | 7.32 | 7.34 | 7.53 | 7.44 | 7.44 |

Table S9. FiO_2_ for all mechanically ventilated subjects at study start, after lung injury was achieved, and at study end.

| **FiO_2_ (%)** | | | | | | | | |
| --- | --- | --- | --- | --- | --- | --- | --- | --- |
| **Study Start** | | | **Lung Injury Achieved** | | | **Study End** | | |
| **MV**  **group** | **MV+TTDN50%**  **group** | **MV+TTDN100%**  **group** | **MV**  **group** | **MV+TTDN50%**  **group** | **MV+TTDN100%**  **group** | **MV**  **group** | **MV+TTDN50%**  **group** | **MV+TTDN100%**  **group** |
| 30 | 30 | 30 | 70 | 30 | 60 | 40 | 30 | 25 |
| 30 | 30 | 30 | 30 | 45 | 70 | 30 | 25 | 30 |
| 30 | 30 | 30 | 40 | 30 | 30 | 30 | 45 | 40 |
| 30 | 30 | 30 | 30 | 40 | 70 | 30 | 30 | 40 |
| 30 | 30 | 30 | 100 | 45 | 30 | 30 | 45 | 40 |
| 30 | 30 | 30 | 60 | 30 | 30 | 30 | 40 | 40 |

Table S10. Respiratory rate for all mechanically ventilated subjects at study start, after lung injury was achieved, and at study end.

| **Respiratory Rate**  **(breaths per minute)** | | | | | | | | |
| --- | --- | --- | --- | --- | --- | --- | --- | --- |
| **Study Start** | | | **Lung Injury Achieved** | | | **Study End** | | |
| **MV**  **group** | **MV+TTDN50%**  **group** | **MV+TTDN100%**  **group** | **MV**  **group** | **MV+TTDN50%**  **group** | **MV+TTDN100%**  **group** | **MV**  **group** | **MV+TTDN50%**  **group** | **MV+TTDN100%**  **group** |
| 20 | 20 | 20 | 20 | 20 | 24 | 22 | 25 | 24 |
| 20 | 20 | 20 | 20 | 20 | 22 | 20 | 18 | 22 |
| 18 | 20 | 20 | 20 | 20 | 23 | 22 | 20 | 23 |
| 18 | 20 | 20 | 18 | 20 | 20 | 22 | 20 | 20 |
| 18 | 20 | 20 | 20 | 22 | 20 | 22 | 22 | 18 |
| 18 | 18 | 20 | 20 | 20 | 22 | 20 | 20 | 19 |

**Intravenous Drug Delivery:**

The total dose of each intravenous drug administered during the studies, including sedatives, normalized by weight to subjects in the MV, MV+PNS50%, and MV+PNS100% groups is shown in Table S11.

Table S11. Summary of total drug dose used during the experiments for the mechanically ventilated groups. All drugs administered are normalized by weight.

| **Total Drug Dose**  **(normalized by weight)** | **MV**  **Median**  **(IQR)** | **MV+PNS50%**  **Median**  **(IQR)** | **MV+PNS100%**  **Median**  **(IQR)** | **Kruskal-Wallis**  **(p-value)** |
| --- | --- | --- | --- | --- |
| **Propofol**  **(mg/kg)** | 49  (48-55) | 55  51-57 | 56  47-58 | ns |
| **Midazolam**  **(mg/kg)** | 70  (68-73) | 69  (67-72) | 69  (67-73) | ns |
| **Fentanyl**  **(µg/kg)** | 390  (360-410) | 414  (384-435) | 405  (397-422) | ns |
| **Ketamine**  **(mg/kg)** | 1300  (1200-2050) | 1400  (950-1700) | 1450  (1175-2025) | ns |
| **Norepinephrine bitartrate (µg/min)** | 2.7  (2.3-3.0) | 2.7  (2.5-3.0) | 2.3  (2.2-2.6) | ns |
| **Phenylephrine**  **(µg/kg)** | 1200  (1175-1600) | 1400  (1175-1600) | 1400  (1200-1650) | ns |
| **Oleic Acid**  **(mg/kg)** | 0.12  (0.07-0.16) | 0.11  (0.08-0.20) | 0.09  (0.06-0.12) | ns |

**Serum Inflammatory Markers (end of study):**

IFN-γ and GM-CSF serum concentrations were significantly different between the groups (Table S12). IL-1α, IL-1β, IL-6, IL-8, IL-10, and TNFα levels were not significantly different between the groups (Table S12).

Table S12. Serum inflammatory marker results for the mechanically ventilated groups. Blood samples were taken at the end of the experiment.

| **Serum inflammatory markers**  **(end of study)** | **Concentration (pg/ml)**  **Median**  **(IQR)** | | | **p-value (Kruskal-Wallis test)** | **p-value**  **(Dunn’s multiple comparison test)** | |
| --- | --- | --- | --- | --- | --- | --- |
|  | **MV**  **(n=6)** | **MV+PNS50%**  **(n=6)** | **MV+PNS100%**  **(n=6)** |  |  |  |
| **IFN-γ** | 1,559.0  (0.0-5727.0) | 944.9  (8.7-2736.0) | 6,937.0  (2,514.0-78,859.0) | 0.0133 | MV vs. MV+PNS50% | ns |
|  |  |  |  |  | MV vs. MV+PNS100% | ns |
|  |  |  |  |  | MV+PNS50% vs. MV+PNS100% | 0.0222 |
| **GM-CSF** | 0.0  (0.0-7.4) | 0.0  (0.0-86.0) | 11.5  (4.9-23.0) | 0.0475 | MV vs. MV+PNS50% | ns |
|  |  |  |  |  | MV vs. MV+PNS100% | 0.0479 |
|  |  |  |  |  | MV+PNS50% vs. MV+PNS100% | ns |
| **IL-1α** | 41.9  (10.0-101.3) | 59.0  (17.9-81.6) | 15.1  (5.3-44.4) | ns | n/a | |
| **IL-1β** | 92.5  (9.2-821.8) | 34.7  (10.1-417.0) | 124.5  (60.3-447.9) | ns | n/a | |
| **IL-6** | 58.2  (32.7-209.1) | 51.7  (22.2-140.1) | 69.6  (54.3-146.8) | ns | n/a | |
| **IL-8** | 19.7  (10.9-114.2) | 62.9  (28.0-152.3) | 44.9  (42.2-48.0) | ns | n/a | |
| **IL-10** | 137.1  (82.2-1370.0) | 113.2  (67.2-585.0) | 194.3  (96.5-727.5) | ns | n/a | |
| **TNFα** | 0.0  (0.0-15.7) | 9.4  (0.0-261.8) | 58.1  (32.5-105.0) | ns | n/a | |

**Gas Exchange Measures and Lung Injury Scores:**

Gas exchange measures at the start, after the achievement of lung injury, and the end of the study are shown in Table S13. Lung injury scores, obtained post-euthanasia, are shown in Table S13.

Table S13. Gas exchange measures at the start, after the achievement of lung injury, and the end of the study. Table also shows lung injury scores for all groups. Subjects from the NV group had arterial blood samples taken once, only, at the beginning of the study. Lung injury score ranges from 0.00 to 1.00.

| **Gas exchange measures and lung injury scores** | **Median**  **(IQR)** | | | **p-value (Kruskal-Wallis test)** | **p-value**  **(Dunn’s multiple comparison test)** | |
| --- | --- | --- | --- | --- | --- | --- |
|  | **MV group**  **(n=6)** | **MV+PNS50% group**  **(n=6)** | **MV+PNS100% group**  **(n=6)** |  |  |  |
| PaO_2_/FiO_2_  ratio,  study start  (mmHg) | 511  (467-543) | 513  (406-536) | 520  (481-531) | 0.8543 | MV vs. MV+PNS50% | --- |
|  |  |  |  |  | MV vs. MV+PNS100% | --- |
|  |  |  |  |  | MV+PNS50% vs. MV+PNS100% | --- |
| PaO_2_/FiO_2_  ratio,  lung injury achieved  (mmHg) | 190  (156-197) | 180  (166-192) | 191  (186-194) | 0.6794 | MV vs. MV+PNS50% | --- |
|  |  |  |  |  | MV vs. MV+PNS100% | --- |
|  |  |  |  |  | MV+PNS50% vs. MV+PNS100% | --- |
| PaO_2_/FiO_2_  ratio,  study end  (mmHg) | 271  (250-389) | 363  (267-430) | 431  (382-490) | 0.0360 | MV vs. MV+PNS50% | --- |
|  |  |  |  |  | MV vs. MV+PNS100% | 0.0386 |
|  |  |  |  |  | MV+PNS50% vs. MV+PNS100% | --- |
| Lung injury score | 0.46  (0.41-0.49) | 0.56  (0.48-0.62) | 0.39  (0.37-0.39) | 0.0003 | MV vs. MV+PNS50% | ns |
|  |  |  |  |  | MV vs. MV+PNS100% | ns |
|  |  |  |  |  | MV+PNS50% vs. MV+PNS100% | ns |

**Respiratory Measurements:**

Respiratory measurements for the mechanically ventilated groups, taken at the beginning and the end of the study are shown in Table S14.

Table S14. Respiratory measurements for the mechanically ventilated groups, taken at the beginning and the end of the study. Esophageal pressure was measured at the end of inspiration. Transpulmonary plateau pressure was measured during the end-inspiratory plateau. Driving pressure was calculated as [end-inspiratory plateau pressure] minus [end-expiratory pressure].

| **Time** | **Measurement** | **Median**  **(IQR)** | | | **p-value**  **(Kruskal-Wallis test)** | **p-value**  **(Dunn’s multiple comparison test)** | |
| --- | --- | --- | --- | --- | --- | --- | --- |
|  |  | **MV**  **group**  **(n=6)** | **MV+PNS50%**  **group**  **(n=6)** | **MV+PNS100%**  **group**  **(n=6)** |  |  |  |
| Baseline | Esophageal pressure (cmH_2_O) | 13  (10-15) | 11  (8-11) | 11  (9-14) | ns | MV vs. MV+PNS50% | --- |
|  |  |  |  |  |  | MV vs. MV+PNS100% | --- |
|  |  |  |  |  |  | MV+PNS50% vs. MV+PNS100% | --- |
|  | Plateau pressure  (cmH_2_O) | 18  (16-19) | 17  (15-19) | 16  (13-16) | ns | MV vs. MV+PNS50% | --- |
|  |  |  |  |  |  | MV vs. MV+PNS100% | --- |
|  |  |  |  |  |  | MV+PNS50% vs. MV+PNS100% | --- |
|  | Transpulmonary driving pressure  (cmH_2_O) | 8  (6-9) | 8  (6-9) | 5  (5-6) | 0.0404 | MV vs. MV+PNS50% | ns |
|  |  |  |  |  |  | MV vs. MV+PNS100% | ns |
|  |  |  |  |  |  | MV+PNS50% vs. MV+PNS100% | ns |
|  | Transpulmonary plateau pressure  (cmH_2_O) | 6  (4-6) | 6  (5-6) | 5  (4-5) | ns | MV vs. MV+PNS50% | --- |
|  |  |  |  |  |  | MV vs. MV+PNS100% | --- |
|  |  |  |  |  |  | MV+PNS50% vs. MV+PNS100% | --- |
| Study End | Esophageal pressure (cmH_2_O) | 11  (10-12) | 9  (8-12) | 10  (7-13) | ns | MV vs. MV+PNS50% | --- |
|  |  |  |  |  |  | MV vs. MV+PNS100% | --- |
|  |  |  |  |  |  | MV+PNS50% vs. MV+PNS100% | --- |
|  | Plateau pressure  (cmH_2_O) | 24  (23-27) | 21  (19-24) | 19  (17-21) | 0.0015 | MV vs. MV+PNS50% | ns |
|  |  |  |  |  |  | MV vs. MV+PNS100% | 0.0380 |
|  |  |  |  |  |  | MV+PNS50% vs. MV+PNS100% | ns |
|  | Transpulmonary driving pressure  (cmH_2_O) | 15  (13-17) | 11  (10-14) | 9  (6-14) | 0.0055 | MV vs. MV+PNS50% | ns |
|  |  |  |  |  |  | MV vs. MV+PNS100% | 0.0008 |
|  |  |  |  |  |  | MV+PNS50% vs. MV+PNS100% | ns |
|  | Transpulmonary plateau pressure  (cmH_2_O) | 13  (12-16) | 12  (9-14) | 8  (5-12) | 0.0520 | MV vs. MV+PNS50% | --- |
|  |  |  |  |  |  | MV vs. MV+PNS100% | --- |
|  |  |  |  |  |  | MV+PNS50% vs. MV+PNS100% | --- |

**Hippocampal Apoptosis, Microglia and Astrocyte Measurements:**

Hippocampal apoptosis, microglia and astrocyte percentages are reported in Table S15.

Table S15. Hippocampal apoptosis, microglia and astrocyte percentages for all mechanically ventilated groups.

| **Brain injury and neuroinflammation outcomes** | **Median**  **(IQR)** | | | **p-value (Kruskal-Wallis test)** | **p-value**  **(Dunn’s multiple comparison test)** | |
| --- | --- | --- | --- | --- | --- | --- |
|  | **MV group**  **(n=6)** | **MV+PNS50% group**  **(n=6)** | **MV+PNS100% group**  **(n=6)** |  |  |  |
| Hippocampal apoptotic cell percentage (%) | 26.5  (18.9-27.9) | 8.5  (7.4-14.3) | 6.7  (4.0-9.0) | 0.0002 | MV vs. MV+PNS50% | ns |
|  |  |  |  |  | MV vs. MV+PNS100% | 0.0341 |
|  |  |  |  |  | MV+PNS50% vs. MV+PNS100% | <0.0001 |
| IBA-1-positive hippocampal cells  (microglia)  (%) | 18.0  (17.0-32.2) | 12.7  (11.6-13.8) | 8.8  (7.7-10.3) | 0.0002 | MV vs. MV+PNS50% | ns |
|  |  |  |  |  | MV vs. MV+PNS100% | 0.0004 |
|  |  |  |  |  | MV+PNS50% vs. MV+PNS100% | 0.0049 |
| GFAP-positive hippocampal cells (reactive astrocytes)  (%) | 17.9  (13.9-24.1) | 12.5  (9.1-15.7) | 9.4  (8.0-10.5) | 0.0085 | MV vs. MV+PNS50% | ns |
|  |  |  |  |  | MV vs. MV+PNS100% | 0.0049 |
|  |  |  |  |  | MV+PNS50% vs. MV+PNS100% | ns |

**References:**

E1. Grasso S, Stripoli T, De Michele M, et al. ARDSnet ventilatory protocol and alveolar hyperinflation: Role of positive end-expiratory pressure. *Am J Respir Crit Care Med*. 2007;176(8):761-767. doi:10.1164/rccm.200702-193OC

E2. Reynolds SC, Meyyappan R, Thakkar V, et al. Mitigation of ventilator-induced diaphragm atrophy by transvenous phrenic nerve stimulation. *Am J Respir Crit Care Med*. 2017;195(3):339-348. doi:10.1164/rccm.201502-0363OC

E3. Voldby AW, Brandstrup B. Fluid therapy in the perioperative setting-A clinical review. *J Intensive Care*. 2016;4(1). doi:10.1186/s40560-016-0154-3

E4. Haga HA, Tevik A, Moerch H. Bispectral index as an indicator of anaesthetic depth during isoflurane anaesthesia in the pig. *Vet Anaesth Analg*. 1999;26(1):3-7. doi:10.1111/j.1467-2995.1999.tb00175.x

E5. Bassi TG, Rohrs EC, Fernandez KC, et al. Transvenous diaphragm neurostimulation mitigates ventilation- associated brain injury. Am J Respir Crit Care Med 2021;204: 1391–1402

E6. Bassi TG, Rohrs E, Fernandez K, et al. Direct brain excision : An easier method to harvest the pig’s brain. *Interdiscip Neurosurg*. 2018;14(May):37-38. doi:10.1016/j.inat.2018.05.010

E7. Bassi, TG, Rohrs, EC, Fernandez, KC et al. Brain injury after 50 hours of lung-protective mechanical ventilation in a preclinical model. *Scientific Reports*. 2021;11(5105). doi:https://doi.org/10.1038/s41598-021-84440-1

E8. Davis BM, Salinas-Navarro M, Cordeiro MF, Moons L, et al. Characterizing microglia activation: A spatial statistics approach to maximize information extraction. *Sci Rep*. 2017;7(1). doi:10.1038/s41598-017-01747-8

E9. Task Force of the European Society of Cardiology the North American Society of Pacing Electrophysiology. Guidelines Heart rate variability. *European Heart Journal*. 1996;17:354-381. doi:10.1161/01.CIR.93.5.1043

E10. Matute-Bello G, Downey G, Moore BB, et al. An official American thoracic society workshop report: Features and measurements of experimental acute lung injury in animals. In: *American Journal of Respiratory Cell and Molecular Biology*. Vol 44. ; 2011:725-738. doi:10.1165/rcmb.2009-0210ST

E11. González-López A, López-Alonso I, Aguirre A, et al. Mechanical ventilation triggers hippocampal apoptosis by vagal and dopaminergic pathways. *Am J Respir Crit Care Med*. 2013;188(6):693-702. doi:10.1164/rccm.201304-0691OC
